# A Giant Adenomatoid Tumor of the Adrenal Gland: A Report of a Rare and Interesting Case

**DOI:** 10.7759/cureus.115173

**Published:** 2026-08-25

**Authors:** Ines Bayar, Takwa Aissaoui, Ahlem Bellalah, Mohamed Helmi Tabka, Sayadi Hanene

**Affiliations:** 1 Department of Endocrinology, Fattouma Bourguiba University Hospital, Monastir, TUN; 2 Department of Pathology, Fattouma Bourguiba University Hospital, Monastir, TUN; 3 Department of Urology, Fattouma Bourguiba University Hospital, Monastir, TUN

**Keywords:** adrenalectomy, adrenal gland, adrenal incidentaloma, cystic adrenal mass, giant adrenal adenomatoid tumor, immunohistochemistry, mesothelial neoplasm

## Abstract

Adenomatoid tumor (AT) is a rare benign mesothelial neoplasm that typically arises in the genital tract. Adrenal involvement is exceptionally uncommon, with fewer than 50 cases reported in the literature. Because of its nonspecific clinical and radiological features, preoperative diagnosis is challenging and malignancy is often suspected.

We report the case of a 48-year-old man in whom a left adrenal mass was incidentally discovered during evaluation for renal colic. Hormonal assessment demonstrated a non-functioning adrenal lesion. Computed tomography and magnetic resonance imaging revealed a large, well-circumscribed 12-cm heterogeneous mass with predominant cystic degeneration, thick septations, and intralesional calcifications, raising suspicion of a malignant adrenal neoplasm. The patient underwent open left adrenalectomy. Gross examination showed a well-defined yellowish tumor with extensive cystic changes and calcifications. Histologically, the lesion consisted of anastomosing cystic spaces lined by bland flattened to cuboidal cells, associated with lymphoid aggregates, fibrosis, calcifications, and focal ossification. Immunohistochemistry demonstrated strong positivity for CK7, calretinin, WT1, and D2-40, confirming mesothelial differentiation, while CD31 and CD34 were negative. These findings established the diagnosis of adrenal AT. The postoperative course was uneventful, and no recurrence has been observed during follow-up.

Adrenal AT is a rare benign lesion that may mimic malignant adrenal neoplasms, particularly when large and cystic. Histopathological and immunohistochemical examination remains essential for definitive diagnosis. Complete surgical excision is curative and associated with an excellent prognosis.

## Introduction

Adenomatoid tumors (AT) are rare benign neoplasms of mesothelial origin, classically described in the genital tract of both men and women, particularly in the epididymis, testicular adnexa, uterus, and fallopian tubes. Extragenital localizations are uncommon and pose a significant diagnostic challenge. The involvement of the adrenal gland is exceptional [1].

Adrenal ATs are typically incidental findings and often mimic other adrenal lesions on imaging because of their nonspecific radiological features. Definitive diagnosis relies on histopathological examination, supported by immunohistochemical positivity for mesothelial markers [2,3].

Recognizing this rare presentation is vital for clinical management. Although patients frequently undergo complete surgical excision due to initial suspicion of malignancy, adrenal ATs follow a completely benign clinical course. To date, there is no documented evidence of recurrence or metastasis. Documenting these rare occurrences expands the differential diagnosis of incidental adrenal masses and helps clinicians avoid unnecessary radical treatments or misclassification [4].

We present a new case of adrenal AT and review the literature, focusing on clinical presentation, radiological findings, pathological features, differential diagnosis, treatment, and prognosis.

## Case presentation

A 48-year-old man with a medical history of type 2 diabetes mellitus (T2DM) and recurrent nephrolithiasis was referred for evaluation of an incidental left adrenal mass. The lesion was discovered during a renal ultrasound performed in the setting of acute renal colic. Computed tomography revealed a large, well-circumscribed left suprarenal mass. The lesion was heterogeneous, predominantly cystic, with a thick wall and internal septations surrounding a central solid component. Contrast enhancement of the solid component was not significant. Intralesional calcifications were present. The mass was closely related to the spleen and splenic vein without evidence of local invasion. The right adrenal gland appeared normal.

**Figure 1:**
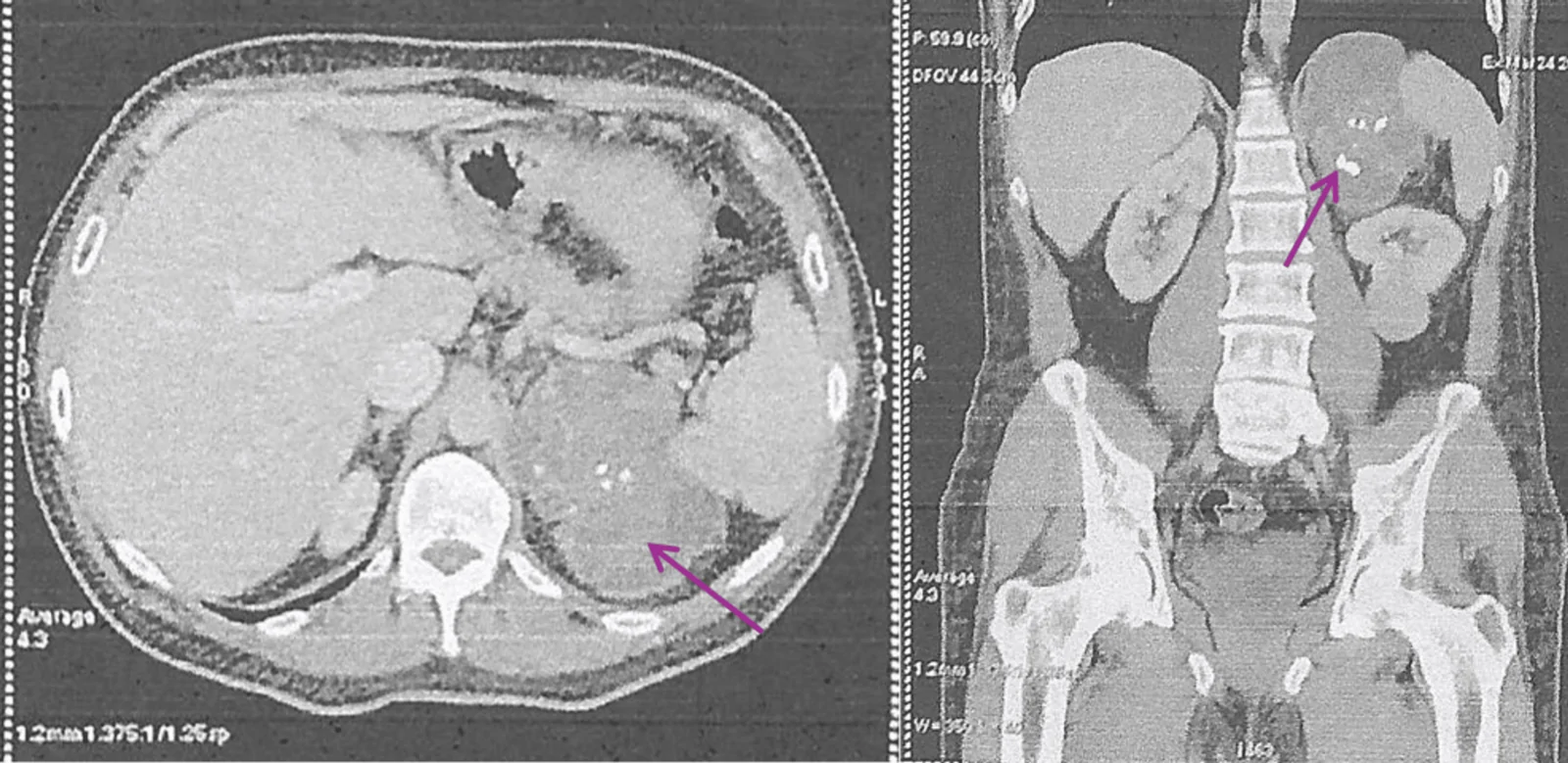
Computed tomography (CT) images (axial and coronal views) demonstrating an adenomatoid tumor of the adrenal gland

Further characterization by contrast-enhanced computed tomography (CT) demonstrated a well-defined left adrenal mass measuring 123 × 105 × 65 mm. The lesion was heterogeneous, predominantly hypodense, with large central non-enhancing cystic-necrotic areas and a thick irregular enhancing peripheral solid component. No macroscopic fat or calcifications were identified. There was no evidence of acute intralesional hemorrhage. The lesion showed heterogeneous contrast enhancement of the viable solid portions without imaging features suggestive of a lipid-rich adrenal adenoma (Figure 1).

Hormonal evaluation revealed a non-functioning adrenal mass. Plasma metanephrines were within normal limits. The patient had no history of hypertension or hypokalemia, and the overnight 1 mg dexamethasone suppression test showed adequate cortisol suppression.

Given the large size of the lesion and its indeterminate radiological features, surgical resection was indicated. The patient underwent open left adrenalectomy through a subcostal approach with perioperative hydrocortisone coverage.

**Figure 2:**
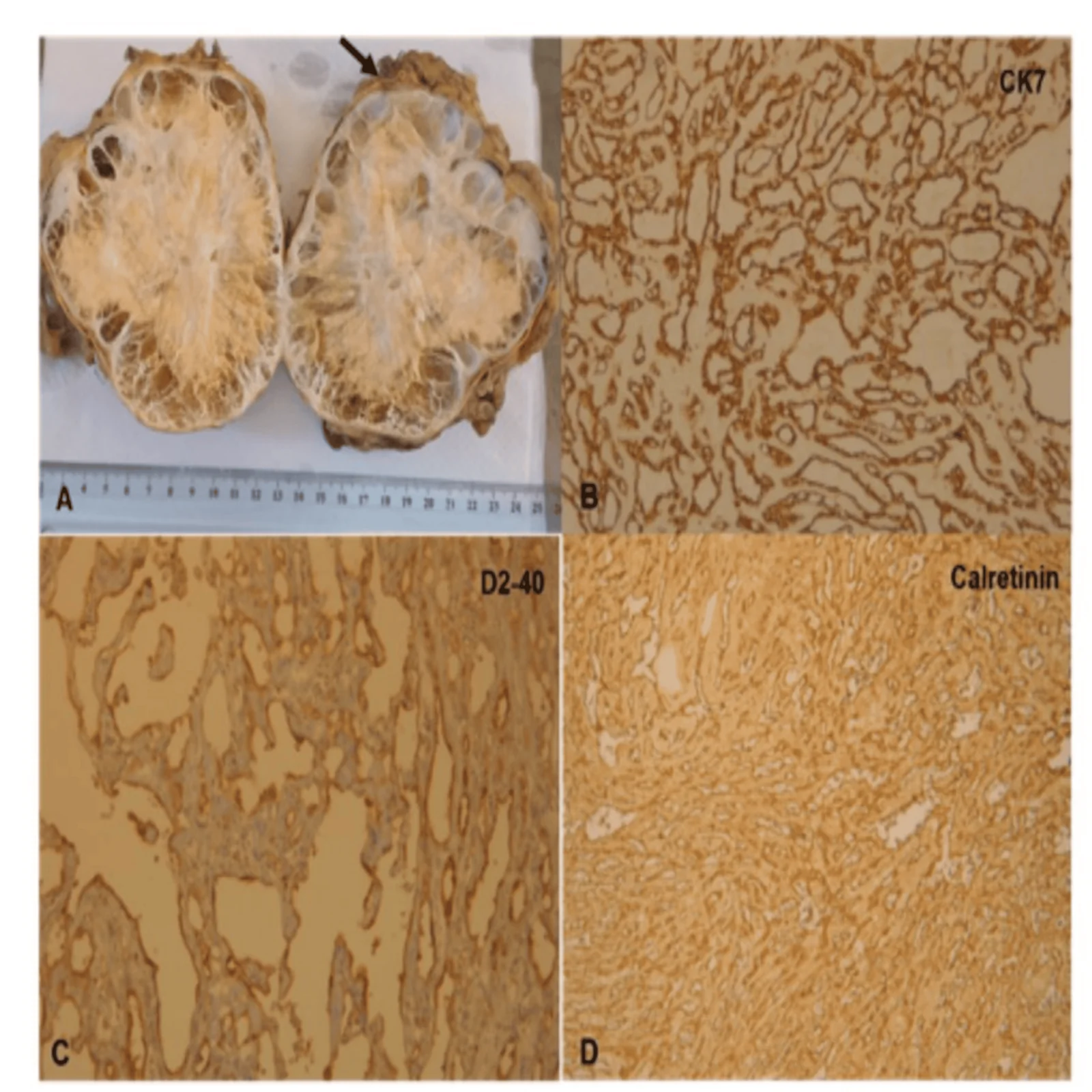
A: Left adrenalectomy specimen showing a yellowish mass of 12 cm with clearly defined borders and solid and spongy aspect. A thin rim of residual adrenal tissue is seen (arrow). The tumor cells show strong positivity with cytokeratin 7 (B, x 100), D2-40 (C, x 100) and calretinin (D, x 40)

Gross examination showed a well-circumscribed yellowish heterogeneous tumor measuring 12 cm in greatest dimension, with extensive cystic changes, a spongy appearance and calcifications. A thin rim of residual adrenal tissue was identified at the tumor margin (Figure 2A).

**Figure 3:**
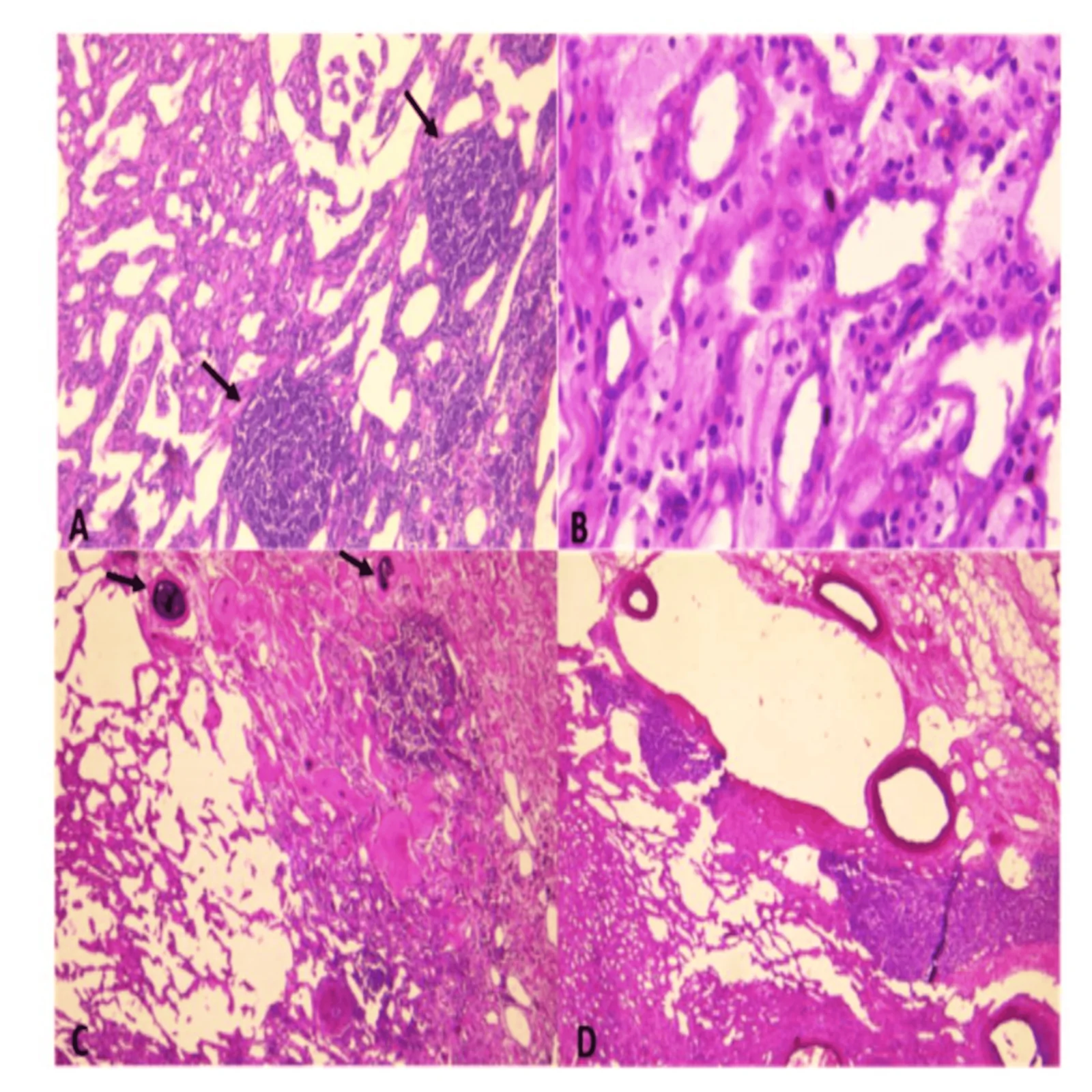
A: The tumor is formed by anastomosing channels separated by lymphoid aggregates (arrows) (HE stain x 100). B: The channels are lined by flattened to hobnailed cells without cytological atypia and surrounded by foamy histiocytes (HE stain x400). C: Dystrophic calcifications are present (arrows) (HE stain x 40). D: The tumor infiltrates the periadrenal adipose tissue (HE stain x 40).

Microscopically, the lesion consisted of variably sized cystic and anastomosing spaces lined by flattened to cuboidal cells. The nuclei were uniform, with inconspicuous nucleoli and no evidence of mitotic activity (Figure 3A, 3B). Numerous lymphoid aggregates and a moderate histiocytic inflammatory infiltrate were present within the stroma. Additional findings included fibrosis, calcifications, and focal ossification (Figure 3C). Focal extension into the periadrenal adipose tissue was observed (Figure 3D).

Immunohistochemical analysis demonstrated strong positivity for cytokeratin 7 (CK7), calretinin, WT1 and D2-40, supporting mesothelial differentiation. Tumor cells were negative for CD31 and CD34, excluding vascular neoplasms. These morphological and immunophenotypic findings established the diagnosis of adrenal AT (Figure 2B-2D).

The postoperative course was uneventful, and the patient remains under clinical and radiological follow-up. At the latest follow-up examination, nine months post-surgery, the patient showed no signs of disease recurrence or hormone imbalance.

## Discussion

ATs are uncommon benign neoplasms of mesothelial origin that predominantly occur in the male and female genital tracts, particularly in the epididymis, testicular adnexa, uterus, and fallopian tubes [5,6]. Extragenital localizations are distinctly uncommon and have been reported in the adrenal gland, pleura, mesentery, pancreas, heart, and lymph nodes [5,6]. Among these unusual sites, adrenal involvement remains exceptionally rare, with fewer than 50 cases reported in the literature to date [3,6].

The pathogenesis of adrenal ATs remains incompletely understood. Their mesothelial origin is now widely accepted based on ultrastructural and immunohistochemical studies demonstrating mesothelial differentiation [6]. The most widely accepted theory suggests that these tumors arise from mesothelial rests entrapped within the adrenal gland during embryogenesis, owing to the intimate developmental relationship between the adrenal cortex and the mesonephric structures of the urogenital ridge [3,7].

Adrenal ATs predominantly affect men during the fourth to sixth decades of life [3,6]. Most lesions are discovered incidentally during imaging investigations performed for unrelated conditions and are generally non-functioning [6,8].

Consistent with these observations, our patient was a 48-year-old man in whom the lesion was detected during the evaluation of renal colic. Hormonal assessment was entirely unremarkable, excluding cortisol, catecholamine, and aldosterone hypersecretion.

Radiological diagnosis remains particularly challenging because no imaging features are pathognomonic. Computed tomography and MRI typically demonstrate well-circumscribed adrenal masses with variable solid and cystic components [3]. Several reports have described heterogeneous lesions containing hemorrhagic, cystic, or fibrotic changes, occasionally associated with calcifications [9]. Consequently, adrenal ATs are frequently mistaken for adrenal cortical adenoma, pheochromocytoma, lymphangioma, adrenal cysts, adrenocortical carcinoma (ACC), or metastatic disease before surgery [3,8].

In the present case, the tumor measured 12 cm and exhibited a complex solid-cystic architecture with thick septations and intralesional calcifications. The considerable size of the lesion and its close relationship with adjacent structures, including the spleen and splenic vein, raised concern for malignancy. Indeed, tumor size remains one of the main factors influencing surgical decision-making in patients with indeterminate adrenal masses [9,10]. The large dimensions observed in our patient place this lesion among the largest adrenal ATs reported in the literature.

Histopathological examination remains the gold standard for the diagnosis of adrenal ATs because neither clinical presentation nor imaging findings are sufficiently specific to establish a definitive diagnosis preoperatively [6,7]. Microscopically, these tumors typically exhibit a combination of adenoid, tubular, angiomatoid, cystic, or solid growth patterns embedded within a fibrous stroma [3,7].

The most characteristic feature is the presence of variably sized interconnected spaces lined by flattened to cuboidal mesothelial cells with bland cytological features and minimal mitotic activity [1,6]. Lymphoid aggregates, stromal fibrosis, vacuolated cells, and focal inflammatory infiltrates have also been frequently described [3,11].

The histological findings observed in our patient were highly consistent with these descriptions. The tumor was composed of multiple anastomosing cystic spaces lined by flattened to cuboidal cells lacking significant atypia or mitotic figures. In addition, numerous lymphoid aggregates, fibrosis, calcifications, and focal ossification were identified. Although focal extension into the periadrenal adipose tissue was present, no histological criteria suggestive of malignancy were observed.

A particularly noteworthy feature in our case was the presence of extensive cystic degeneration associated with calcifications and ossification. Similar findings have occasionally been reported in large adrenal ATs, and may contribute to an erroneous radiological suspicion of ACC or other malignant adrenal neoplasms [7].

Immunohistochemistry plays a pivotal role in confirming the mesothelial nature of these tumors and excluding their numerous histological mimickers [3,6]. Adrenal ATs typically express mesothelial markers such as calretinin, WT1, D2-40, HBME-1, and broad-spectrum cytokeratins [6].

In the present case, tumor cells showed strong positivity for CK7, calretinin, WT1 and D2-40, supporting mesothelial differentiation. The absence of CD31 and CD34 expression excluded a vascular neoplasm, particularly lymphangioma, which represents one of the most differential diagnoses of multicystic adrenal lesions [3].

The immunophenotypic profile observed in our patient is therefore entirely consistent with previously reported adrenal ATs and provides strong evidence supporting the diagnosis [1].

The differential diagnosis of adrenal ATs is broad and includes both benign and malignant adrenal lesions. Because these tumors frequently present as large heterogeneous masses with cystic degeneration, establishing a preoperative diagnosis is often impossible [3,10].

Among benign lesions, adrenal lymphangioma represents one of the principal histological mimickers. Both entities may exhibit multicystic architecture and can be associated with calcifications. However, lymphangiomas are composed of endothelial-lined vascular spaces and typically express endothelial markers such as CD31 and CD34 while lacking mesothelial marker expression [3].

Adrenal endothelial cysts should also be considered in the differential diagnosis of predominantly cystic adrenal masses. Unlike ATs, endothelial cysts generally lack the characteristic anastomosing architecture and mesothelial immunophenotype observed in the present case [12].

From a radiological perspective, large ATs may mimic ACC, particularly when they exceed 6 cm, display heterogeneous enhancement, contain necrotic or cystic areas, or exhibit calcifications [3]. In the present case, the tumor measured 12 cm and demonstrated extensive cystic degeneration with thick septations and calcifications, findings that strongly raised suspicion of malignancy before surgery.

Metastatic carcinoma may also enter the differential diagnosis, especially when glandular or pseudovascular patterns are prominent microscopically. Nevertheless, metastatic lesions generally demonstrate significant cytological atypia and lack expression of mesothelial markers such as calretinin and D2-40 [7].

Malignant mesothelioma represents another consideration because both lesions share a mesothelial origin and may express similar immunohistochemical markers. However, mesothelioma is characterized by infiltrative growth, marked cytological atypia, increased mitotic activity, and aggressive clinical behavior, features that were entirely absent in our patient [7,12].

Therefore, accurate diagnosis relies on careful integration of morphology and immunohistochemical findings. The combination of bland cytological features, characteristic anastomosing cystic spaces, positivity for CK7, calretinin, and D2-40, and negativity for endothelial and melanocytic markers strongly supported the diagnosis of adrenal AT in our case.

Given the absence of specific clinical, biochemical, or radiological findings, adrenal ATs are rarely diagnosed before surgery [3,6]. Consequently, most reported patients undergo adrenalectomy because the possibility of ACC or another malignant adrenal neoplasm cannot be confidently excluded preoperatively [3,7].

Current international guidelines recommend surgical resection for adrenal masses exhibiting suspicious imaging characteristics, hormonal activity, or large size, particularly when malignancy cannot be ruled out [9]. In the present case, several factors supported surgical management, including the large tumor size (12 cm), heterogeneous solid-cystic appearance, thick septations, intralesional calcifications, and indeterminate radiological features. Therefore, open adrenalectomy was considered the most appropriate therapeutic approach.

Complete surgical excision is both diagnostic and curative for adrenal ATs [3]. Histopathological examination remains essential for establishing the definitive diagnosis and excluding malignant lesions.

Despite occasional reports of focal extension into surrounding adipose tissue, adrenal ATs consistently demonstrate benign biological behavior [5,7]. To date, no cases of metastatic dissemination or malignant transformation have been documented in the literature following complete surgical resection [3,6].

The prognosis is therefore excellent, and long-term follow-up data available from published cases have not identified tumor recurrence after complete excision [3]. Nevertheless, periodic clinical and radiological surveillance may be considered, particularly in patients with large tumors or limited postoperative follow-up. Our patient remains under regular follow-up and has shown no evidence of recurrence to date.

To better contextualize our findings, the clinicopathological characteristics of previously reported adrenal ATs are summarized in Table 1. Compared with published cases, our patient presented with one of the largest tumors reported to date, characterized by extensive cystic degeneration, calcifications, and focal ossification.

**Table 1:** Summary of clinicopathological features of adrenal adenomatoid tumors reported in the literature and comparison with the present case. Data extracted from [1,2,4,6,8].

Feature	Previously reported adrenal adenomatoid tumors	Present case
Number of reported cases	Fewer than 50 cases reported in the English-language literature	One additional case
Age	22-64 years; mean age approximately 39 years	48 years
Sex predominance	Marked male predominance	Male
Laterality	Both adrenal glands may be affected, with no clear side predominance	Left adrenal gland
Mode of discovery	Mostly incidental radiological, surgical, or autopsy findings	Incidental finding during renal ultrasound for acute renal colic
Symptoms	Usually asymptomatic; occasionally associated with hypertension, nephrolithiasis, abdominal pain, hematuria, or other unrelated conditions	Recurrent nephrolithiasis; no adrenal-related symptoms
Hormonal status	Usually non-functioning	Non-functioning; normal metanephrines and adequate cortisol suppression after 1-mg dexamethasone test
Tumor size	0.5-19 cm; mean approximately 5.3 cm	12 cm
Gross appearance	Usually well-circumscribed, gray-yellow or yellowish mass; solid, solid-cystic, or rarely entirely cystic	Well-circumscribed yellowish heterogeneous mass with extensive cystic degeneration, calcifications, and spongy peripheral appearance
Imaging features	Non-specific; may appear solid, cystic, or mixed solid-cystic; may mimic adrenal adenoma, cyst, lymphangioma, pheochromocytoma, or malignancy	Large heterogeneous solid-cystic mass with thick septations, central solid component, intralesional calcifications, and no macroscopic fat
Histological patterns	Adenoid, angiomatoid, tubular, cystic, papillary, or solid patterns; often mixed	Anastomosing cystic spaces lined by bland flattened to cuboidal cells
Cytological atypia/mitoses	Usually absent	Absent
Necrosis	Usually absent	Absent
Lymphoid aggregates	Frequently reported	Present
Calcifications/ossification	Calcifications occasionally reported; ossification rarely described	Calcifications and focal ossification present
Local extension	Extension into adrenal cortex, medulla, capsule, or periadrenal adipose tissue may be seen and is not considered a criterion of malignancy	Focal extension into periadrenal adipose tissue
Positive immunohistochemical markers	Cytokeratins, CK7, calretinin, D2-40, WT1, HBME-1	CK7, calretinin, WT1, D2-40
Negative immunohistochemical markers	CD31, CD34, HMB45, Melan-A, actin, desmin, synaptophysin, chromogranin are usually negative	CD31, CD34, HMB45, AML, desmin
Treatment	Surgical excision, usually adrenalectomy	Open left adrenalectomy
Prognosis	Excellent; no recurrence, metastasis, or malignant transformation reported after complete excision	Under follow-up; no recurrence to date
Particular interest	Rare benign mesothelial tumor with non-specific imaging features	Unusually large 12-cm tumor with solid-cystic morphology, focal ossification, and radiological suspicion of malignancy

Similar to most previously reported cases, our patient was a middle-aged man with a non-functioning adrenal mass and an excellent postoperative outcome. However, the large size of the lesion and its complex radiological appearance contributed to a strong preoperative suspicion of malignancy.

## Conclusions

Adrenal AT is an exceptionally rare benign mesothelial neoplasm that should be considered in the differential diagnosis of large non-functioning adrenal masses. Because its clinical, biochemical, and radiological features are non-specific, preoperative diagnosis remains challenging and malignancy is frequently suspected. Histopathological examination combined with immunohistochemical analysis is essential for establishing the diagnosis and excluding other cystic or malignant adrenal lesions. Our case highlights an unusually large adrenal AT with extensive cystic degeneration, calcifications, and focal ossification, radiological features that closely mimicked a malignant adrenal neoplasm. Complete surgical excision remains both diagnostic and curative, and the prognosis appears excellent with no reported cases of recurrence or malignant transformation following complete resection.
